# Striatal Serotonin 4 Receptor is Increased in Experimental Parkinsonism and Dyskinesia

**DOI:** 10.3233/JPD-230331

**Published:** 2024-03-05

**Authors:** Rossella Cirillo, Sandra Duperrier, Pathik Parekh, Mathilde Millot, Qin Li, Marie-Laure Thiolat, Micaela Morelli, Jing Xie, Didier Le Bars, Jérôme Redouté, Erwan Bezard, Véronique Sgambato

**Affiliations:** a Institut des Sciences Cognitives Marc Jeannerod (ISCMJ), Unité Mixte de Recherche 5229 du Centre National de la Recherche Scientifique (CNRS), Bron, France; b Universitè de Lyon 1, Lyon, France; c Department of Biomedical Sciences, Section of Neuroscience, University of Cagliari, Monserrato, Italy; d Motac Beijing Services, Beijing, China; e Universitè de Bordeaux, Institut des Maladies Neurodégénératives, Bordeaux, France; f CNRS, Institut des Maladies Neurodégénératives, Bordeaux, France; g Institut du Vieillissement, Centrede Recherche Clinique Vieillissement Cerveau Fragilité, Hôpital des Charpennes, Villeurbanne, France; h CERMEP-Imagerie du Vivant, Lyon, France

**Keywords:** Parkinson’s disease, serotonin, PET imaging, immunohistochemistry, experimental animal models, L-Dopa, movement disorders, parkinsonism, dyskinesia

## Abstract

Alterations of serotonin type 4 receptor levels are linked to mood disorders and cognitive deficits in several conditions. However, few studies have investigated 5-HT_4_R alterations in movement disorders. We wondered whether striatal 5-HT_4_R expression is altered in experimental parkinsonism. We used a brain bank tissue from a rat and a macaque model of Parkinson’s disease (PD). We then investigated its *in vivo* PET imaging regulation in a cohort of macaques. Dopaminergic depletion increases striatal 5-HT_4_R in the two models, further augmented after dyskinesia-inducing L-Dopa. Pending confirmation in PD patients, the 5-HT_4_R might offer a therapeutic target for dampening PD’s symptoms.

## INTRODUCTION

Parkinson’s disease (PD) is characterized by the loss of dopaminergic (DA) neurons in the substantia nigra, leading to cardinal motor symptoms, bradykinesia, akinesia, rigidity, resting tremor and postural abnormalities [1]. The neurodegenerative process also affects the serotonergic (5-HT) neurons in raphe nuclei [2]. Strong links have been established between the alteration of the presynaptic 5-HT system (5-HT transporter, 5-HT_1A_/_2A_ receptors) and manifestations of tremor, levodopa-induced dyskinesias (LIDs) and neuropsychiatric symptoms [3, 4].

Beyond the presynaptic 5-HT system, there is a growing interest towards the post-synaptic serotonin 4 receptor (5-HT_4_R) [5, 6]. This G-protein coupled receptor is widely distributed in the body and highly expressed in the brain, especially in the basal ganglia. Its activation modulates food intake [7] and supports pro-cognitive, anxiolytic and antidepressant effects [8, 9]. 5-HT_4_R agonists treat chronic idiopathic constipation in humans [10] and improve memory [11]. 5-HT_4_R expression is knowingly altered in abnormal food intake, mood disorders and cognitive deficits [12–14].

Surprisingly few studies have focused on the 5-HT_4_R in PD, while the myriad of PD non-motor symptoms encompasses such manifestations [15, 16]. As a first step, we wondered whether the striatal 5-HT_4_R is increased after DA depletion and L-Dopa supplementation using an existing brain bank tissue from a rat and a non-human primate (NHP) model of PD. We then investigated its *in vivo* PET imaging regulation in a second cohort of NHPs.

## MATERIALS AND METHODS

### Animals

Experiments were carried out in accordance with European Communities Council Directive of November 24, 1986 (86/609/EEC) revised in 2010 (2010/63/UE) and were approved by the local ethical committees. Following the three Rs (Reduction, Refinement, and Replacement) for animal experimentation, we first used existing well-validated brain collections featuring parkinsonian and dyskinetic rats [17] and *Macaca mulatta* NHPs [18]. Briefly, rats were rendered hemiparkinsonian following unilateral injection of 6-hydroxydopamine (12μg) into the substantia nigra pars compacta, rendered dyskinetic by a 10 days-treatment with L-Dopa (25 mg/kg twice daily), and sacrificed 6 weeks after dopaminergic lesion [17]. *Macaca mulatta* NHPs were rendered parkinsonian by daily injection of 1-methyl 4-phenyl 1,2,3,6-tetrahydropyridine (MPTP at 0.2 mg/kg/day) until stabilization of parkinsonian symptoms, dyskinetic by a 3-months oral L-Dopa treatment (20 mg/kg twice daily), and were sacrificed 6 months after onset of DA lesion [18]. For both species, behavioral analysis and lesion characteristics were published in detail [17, 18]. However, parkinsonian and dyskinetic measures obtained for rats and macaques are indicated in Supplementary Table 1. These measures have been acquired through the use of specific, well-known tests and scales (the rotational behavior and the scoring of axial, orolingual and forelimb dyskinesia [19] for rats; the PD disability score [20], the monkey clinical assessment scale [21] and the NHP dyskinesia rating scale [22] for macaques). *In vivo* molecular imaging was conducted on a novel macaque cohort, the experimental details of which are given below.

### Postmortem studies

Brain collections were issued from 15 rats (4 control, 5 hemiparkinsonian, 6 dyskinetic) and 11 macaques (4 controls, 4 parkinsonian, 3 dyskinetic). For each animal, four sections at the level of the posterior striatum were processed for immunohistochemistry (see detailed protocol in [23]) with the following antibodies: anti-5-HT_4_ receptor (5-HT_4_R) 1/100 rabbit polyclonal from Merck (Merck, Molsheim, France) (catalog number S0195), anti-FosB/*Δ*FosB 1/200 rabbit polyclonal from Tebu-Bio (catalog number SC-7203; Tebu-Bio, Le Perray en Yvelines, France). The specificity of the immunostaining was assessed by omission of the primary antibodies from the protocol. At the end of the protocol, sections were examined with a light microscope using a computerized image analyzer (Mercator, ExploraNova, La Rochelle, France). Striatal 5-HT_4_R expression levels were analyzed under blinded conditions relative to the animal by optical density measurements using Image J software.

### In vivo molecular imaging studies

Six adult male macaques (*Macaca fascicularis*) were used for *in vivo* molecular imaging. Monkeys weighed between 4 and 9 kg and were aged between 4 and 6 years. They were kept under standard conditions (12 h light cycles, 23°C, and 50% humidity). They were rendered parkinsonian by systemic intoxication with MPTP (0.4 mg/kg) (MPTP from Sigma-Aldrich, Saint-Quentin-Fallavier, France). MPTP injections were stopped once Parkinsonian symptoms were established, as previously described [24]. The six monkeys were scanned before (baseline) and two months after MPTP intoxication (post-MPTP) with [^11^C]PE2I and [^11^C]SB207145, which bind to the dopaminergic transporter [25] and the 5-HT_4_ receptor [26, 27], respectively. PET (positron emission tomography) and MRI (magnetic resonance imaging) acquisitions were performed at the imaging center (CERMEP, Lyon, France) under anesthesia (atropine 0.05 mg/kg intramuscularly followed 15 min later by zoletil 15 mg/kg intramuscularly). Anatomical MRI acquisition consisted of a 3D T1-weighted sequence using a 1.5-T AvantoFit scanner (Siemens). The anatomical volume covered the whole brain with 176 planes of 0.6 mm cubic voxels. PET imaging was performed using a Siemens Biograph mCT/S64 scanner. The Biograph mCT had a spatial transverse resolution of 4.4 mm. Attenuation was obtained using a 1 min low-dose CT scan acquired before emission. Dynamic acquisition started with the intravenous injection of the radiotracer, synthesized in the cyclotron unit at CERMEP, and lasted 90 min for SB207145 scans and 70 min for PE2I scans. PET emission images were corrected for attenuation, random and scatter and reconstructed using the Siemens ultraHD PET algorithm with 12 iterations, 21 subsets and a zoom factor of 8. Reconstructed volumes were 109 slices (2.027 mm thickness, 256×256 matrices of 0.398×0.398 mm2 voxels), and consisted in multi-frames of increasing durations ([^11^C]SB207145:4×30 s, 6×60 s, 9×180 s, 11×300 s; [^11^C]PE2I: 4×30 s, 4×60 s, 8×180 s, 8×300). Individual PET images were registered to their corresponding individual anatomical MRI, which was registered to the *Macaca fascicularis* MRI template [28]. Transformations from native PET to individual MRI and individual MRI to template were then concatenated to provide direct (and inverse) affine transformations from PET native spaces to the template space. PET data were analyzed by tracer kinetic modelling at a voxel-based level. The parameters computed were the non-displaceable binding potential (BP_ND_) of [^11^C]SB207145 and of [^11^C]PE2I using a simplified reference tissue model. The cerebellum (excluding the vermis) was considered as the reference region for the modelisation. Regional values of BP_ND_ were extracted from parametric maps using MAXPROB atlas as described in [24].

### Statistical analysis

All statistical analyses were performed using GraphPad Prism software. PET imaging and immunohistochemical data were analyzed using non-parametric Mann–Whitney tests with *p* < 0.05. Histograms represent mean±SEM.

## RESULTS

**Fig. 1:**
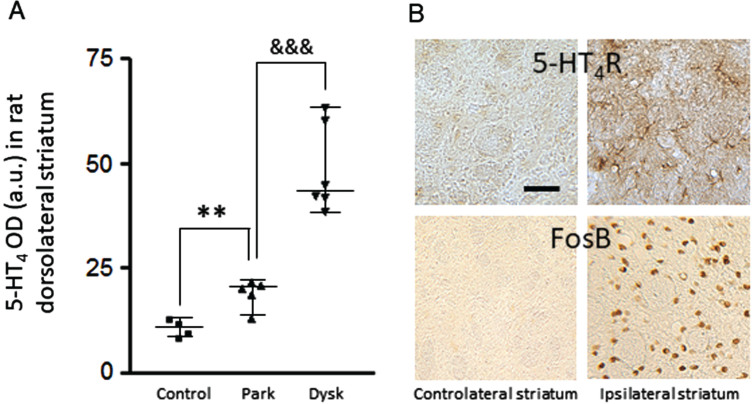
Striatal upregulation of 5-HT_4_R after dopamine depletion and L-Dopa exposure in the 6-OHDA lesioned rat. A) Histogram represents optical density measurements of 5-HT_4_R (in arbitrary units) in the ipsilateral dorsolateral striatum for the 3 experimental groups. **p < 0.01 versus control; ^&&&^*p* < 0.001 versus parkinsonian. B) Photomicrographs at high magnification (x16) of coronal sections of a dyskinetic rat, exemplifying spatio-temporal concomitance of 5-HT_4_R and FosB increases in the ipsilateral dorsolateral striatum. Scale bar on B is 50μm. OD, optical density, Park, parkinsonian; Dysk, dyskinetic.

5-HT_4_R upregulated in the dorsolateral striatum of dopamine-depleted 6-hydroxydopamine (6-OHDA) rats (Fig. 1A). An even greater upregulation was induced by L-Dopa chronic exposure of these unilaterally lesioned rats (Fig. 1A, B). The neuropil was densely labelled, revealing some dendritic branches and varicosities (Fig. 1B). Of note is the observation of a spatial coincidence between the striatal upregulation of 5-HT_4_R and the striatal increase in FosB/*Δ*FosB, a transcriptional factor critically involved in L-Dopa-induced dyskinesia pathophysiology [29] (Fig. 1B).

**Fig. 2:**
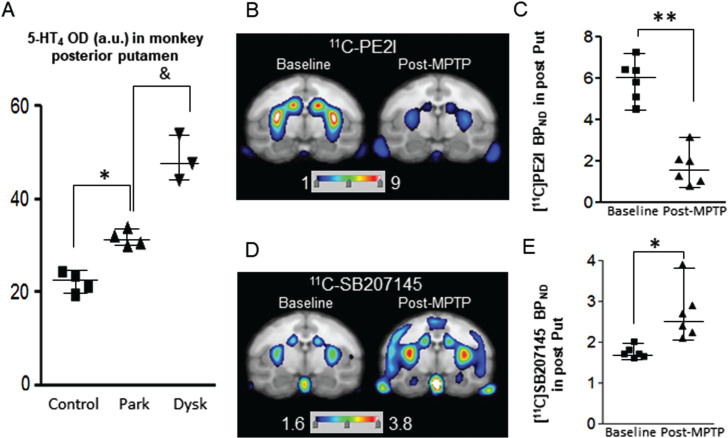
Striatal upregulation of 5-HT_4_R after dopamine depletion and L-Dopa exposure in MPTP-treated macaques. A) Histogram represents optical density measurements of 5-HT_4_R (in arbitrary units) in the posterior putamen for the 3 experimental groups. *p < 0.05 versus control; ^&^*p* < 0.05 versus parkinsonian. B) [^11^C]PE2I PET averaged images (in color) on coronal planes at the level of the posterior caudate and putamen for each condition. C) Histogram represents ^11^C-PE2I BP_ND_ in the posterior putamen for each condition. D) [^11^C]SB207145 PET averaged images (in color) on coronal planes at the level of the posterior caudate and putamen for each condition. E) Histogram represents ^11^C-SB207145 BP_ND_ in the posterior putamen for each condition. *p < 0.05, **p < 0.01 versus baseline. Color represents the level of BP_ND_ using the cerebellum as the reference region (red indicates high whereas bleu indicates low BP_ND_ on each scale). a.u., arbitrary units; BP_ND_, non-displaceable binding potential; Dysk, dyskinetic; OD, optical density; Park, parkinsonian.

Such 5-HT_4_R upregulation was also observed in the gold-standard experimental model of PD, namely the MPTP-treated macaque NHP (Fig. 2A). Chronic (3 months) L-Dopa supplementation at therapeutic doses led to LID manifestations and to further upregulation of striatal 5-HT_4_R levels (Fig. 2A).

Given the translational value of Parkinsonian macaques, we then longitudinally investigated 5-HT_4_R *in vivo* binding by PET imaging before (control situation) and after MPTP intoxication (Parkinsonian situation). The extent of nigrostriatal lesion was documented using a clinical-grade radiotracer specific to the dopamine transporter, the [^11^C]PE2I (Fig. 2B, C). We then ran [^11^C]SB207245, a highly specific radiotracer of 5-HT_4_R [30]. [^11^C]SB207145 BP_ND_ was increased after parkinsonism induction in all striatal areas (Fig. 2D), notably in the posterior motor putamen (Fig. 2E).

## DISCUSSION

This study shows that dopaminergic depletion is sufficient to induce a striatal upregulation of the 5-HT_4_R, and that this increase is potentiated, and concomitant with FosB/*Δ*FosB, following L-Dopa supplementation causing dyskinesias.

5-HT_4_R distribution in the brain is highly conserved across species [31, 32]. Very few studies investigated 5-HT_4_R expression regulation so far. Experimentally, they were performed only in rodents, i.e., rats and guinea pigs. In the 6-OHDA-injured rat, Compan and colleagues (1996) showed a 59% increase in 5-HT_4_R binding in the caudal part of the caudate-putamen [33]. A recent *in situ* hybridization study, therefore measuring mRNA transcripts and not receptors themselves, did not detect changes in striatal 5-HT_4_R mRNA levels after dopaminergic lesion or after chronic L-Dopa treatment [34]. These results suggest that the 5-HT_4_R expression must be functionally investigated with direct binding or immunohistochemistry, as 5-HT_4_R displays a commonly observed decoupling between the transcript abundance and the protein expression.

Only two studies, from the same lab in 1995, report tritiated radioligand binding studies of the 5-HT_4_R in postmortem human brain homogenates. Peculiarly, the authors did not report a difference in putaminal 5-HT_4_ binding levels between control and Parkinsonian subjects although the mean binding values were increased [35, 36]. The low power of the studies associated to the lack of spatial resolution due to the homogenization of tissues as opposed to ligand binding or immunostaining of brain sections should account for this difference. The trend is however similar.

In conclusion, the 5-HT_4_R is over-expressed in the putamen both after DA depletion and DA dyskinesiogenic supplementation. This suggests that two distinct mechanisms are involved: firstly, a post-injury compensatory mechanism, and secondly, a LID-driven maladaptive plasticity mechanism involving FosB/*Δ*FosB, which may in turn regulate the transcription of 5-HT_4_R. Indeed, *Δ*FosB likely binds to the 5-HT_4_R [37]. Although we do not know, at this stage, whether 5-HT_4_R is involved in motor disorders or is due to compensatory mechanisms, this work raises the broader question of the role of the 5-HT_4_R in the pathophysiology of PD, with possible implications on the pathophysiology of cognitive deficits or mood disorders to which this receptor has been linked in other pathologies. Future PET imaging studies in humans urgently need to confirm (or infirm) these preclinical results since the 5-HT_4_R upregulation might offer a therapeutic target for dampening PD’s motor symptomatology. However, the clinical use of 5-HT_4_ antagonists could prove tricky given the lack of available pharmacological agents and the high risk of inducing non-targeted side effects, particularly due to the expression of these receptors outside the brain, such as in the gastrointestinal tract or the heart [5, 38]. Also, given that constipation [39, 40] and non-motor disorders [41, 42] are reportedly improved by 5-HT_4_R agonists, the neurologists may have to opt for different pharmacological options to treat these non-motor symptoms alongside motor disorders.

## Supplementary Material

Supplementary Material

## Data Availability

The data supporting the findings of this study are available on request from the corresponding author.
