# Supplementary material for: Striatal Serotonin 4 Receptor is Increased in Experimental Parkinsonism and Dyskinesia
Source: J Parkinsons Dis. 2024 Mar 5;14(2):261–7. doi: 10.3233/JPD-230331 (PMC10977406; doi:10.3233/JPD-230331)
Supplement: Supplementary Material [file jpd-14-jpd230331-s001.pdf]

Supplementary Material

Striatal Serotonin 4 Receptor Is Increased in Experimental Parkinsonism and Dyskinesia

Supplementary Table 1. Scoring of parkinsonism and dyskinesia for both rats and macaques

		Brain-bank tissue				*In vivo* PET imaging	
	Number of animals	Rats		Macaques		Macaques	
		Number of rotations / min	Dyskinetic score (0 to 12)	Disability score (0 to 10)	Dyskinetic score (0 to 4)	Disability score (0 to 10)	Parkinsonian score (0 to 29)
Controls	1	0	0	0	0	0	0
	2	0	0	0	0	0	0
	3	0	0	0	0	0	0
	4	0	0	0	0	0	0
	5					0	0
	6					0	0
DA-depleted	1	10	0	9	0	7	19
	2	12	0	7	0	8	21
	3	7	0	10	0	8	21
	4	9	0	8	0	10	24
	5	6	0			10	24
	6					9	22
DA-depleted and L-DOPA-treated	1	7	8	9	2		
	2	12	9	10	3		
	3	15	10	8	3		
	4	10	9				
	5	5	6				
	6	5	7				
